# Intramedullary Plate Fixation and Viable Bone Allografting in a Complex Intra-articular Distal Radius Fracture Nonunion: A Case Report

**DOI:** 10.7759/cureus.57308

**Published:** 2024-03-31

**Authors:** Neil Jain, Evan Hernandez, Yasier Medina, Caleb Gottlich, Brendan J MacKay

**Affiliations:** 1 Department of Orthopaedic Surgery, Texas Tech University Health Sciences Center, Lubbock, USA

**Keywords:** viable bone allograft, #distal radius fracture, #nonunion, intramedullary fragment plate, dorsal spanning plate, cancellous bone chips

## Abstract

In this report, we detail a 69-year-old female who sustained a comminuted intra-articular left distal radius fracture that failed to heal with bridge plate fixation. Given the patient’s poor subchondral bone stock and refusal of bone autograft, we designed a construct using a dorsal spanning plate and an intramedullary fragment-specific plate as a volarly placed strut in combination with viable bone allograft and cancellous bone chips to treat this nonunion. This case demonstrates an option for distal radius non-union treatment and highlights the importance of ingenuity that orthopedic surgeons should demonstrate when trying to accommodate patients’ wishes.

## Introduction

Distal radius fractures are the most common type of bony injuries seen in adults [1]. They occur in a bimodal distribution, usually involving those younger than 18 years or older than 50 with osteopenia or osteoporosis [2]. The gold standard for management is volar locking plate fixation with fragment-specific treatment indicated when the articular surface is severely comminuted [3-5]. As an alternative, bridge plating involving temporary placement of a dorsal spanning plate (DSP) has been favorably described for maintaining radial length, distracting the articular surface, and providing rigid stabilization to prevent articular surface loading [6,7].

Nonunion of distal radius fractures is an uncommon occurrence with reported rates ranging from 0.03-0.2% of cases [8-11]. Given the rarity of this complication, there has been no consensus on the best approach to management. Examples of treatment options include DSP application, resection of the site of nonunion followed by internal fixation and bone grafting, or wrist arthrodesis in cases where there is not 5 mm of subchondral bone stock available beneath the lunate facet of the distal radius for application of an implant [8,10-12].

In this report, we describe a case of an intra-articular left distal radius fracture that progressed to nonunion after bridge plating. The workup included radiographs and laboratory testing to evaluate for metabolic, endocrine, and infectious causes. The patient’s inflammatory markers, cell counts, and hemoglobin A1c were within normal limits, and only a significant finding of osteopenia was treated with Vitamin D and calcium. Given the patient’s poor subchondral bone stock that would not support repair using a volar plate and refusal of bone autograft, we designed a construct using a dorsal spanning plate and an intramedullary fragment-specific plate as a volarly placed strut in combination with viable bone allograft (VBA) and cancellous bone chips to treat this complicated nonunion [13-15].

## Case presentation

**Figure 1:**
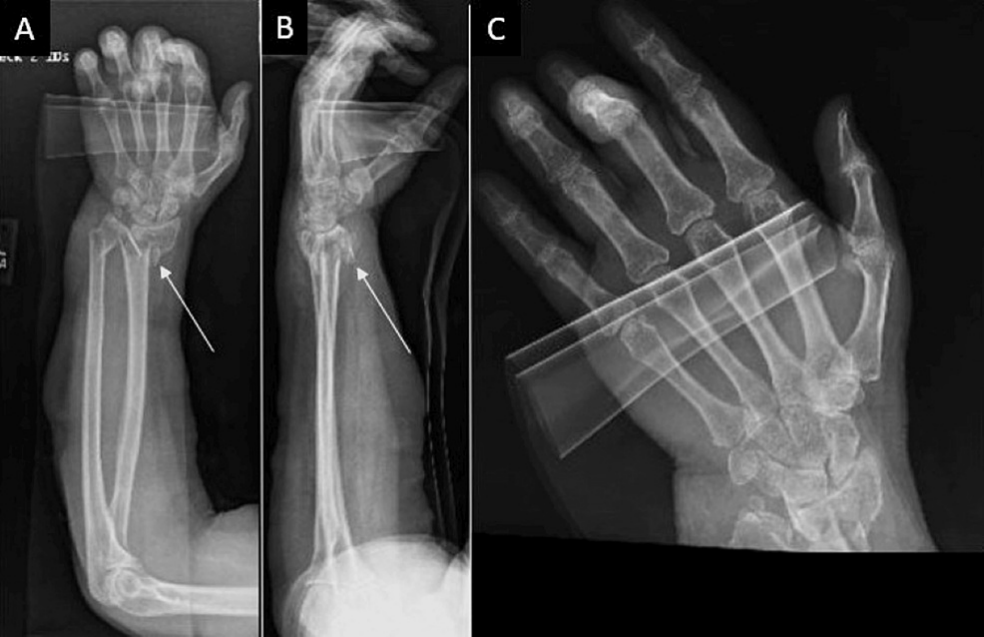
Initial presentation of the comminuted left distal radius fracture A: AP view; B: Lateral view; C: Hand and wrist films

**Figure 2:**
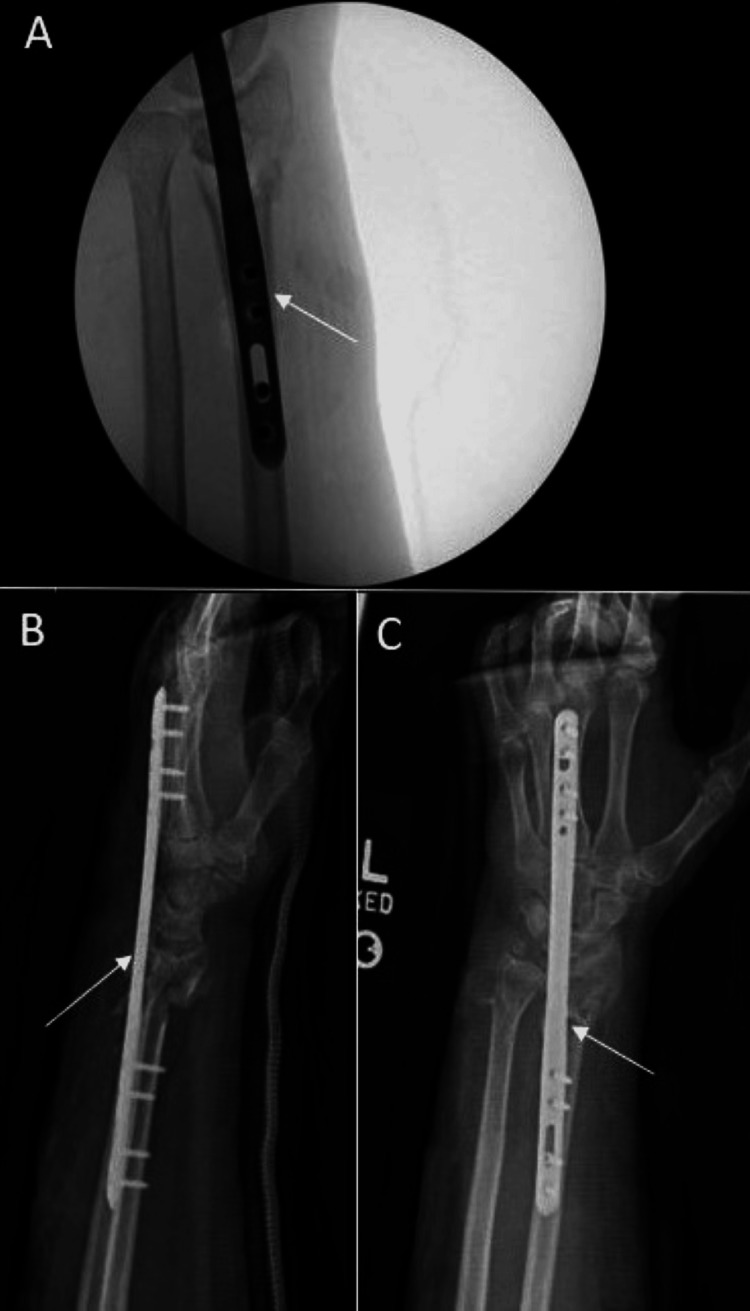
First fixation using a Skeletal Dynamics short distal radius spanning plate A: Intraoperative fluoroscopy of plate placement B/C: Complete views of dorsal spanning plate positioning

**Figure 3:**
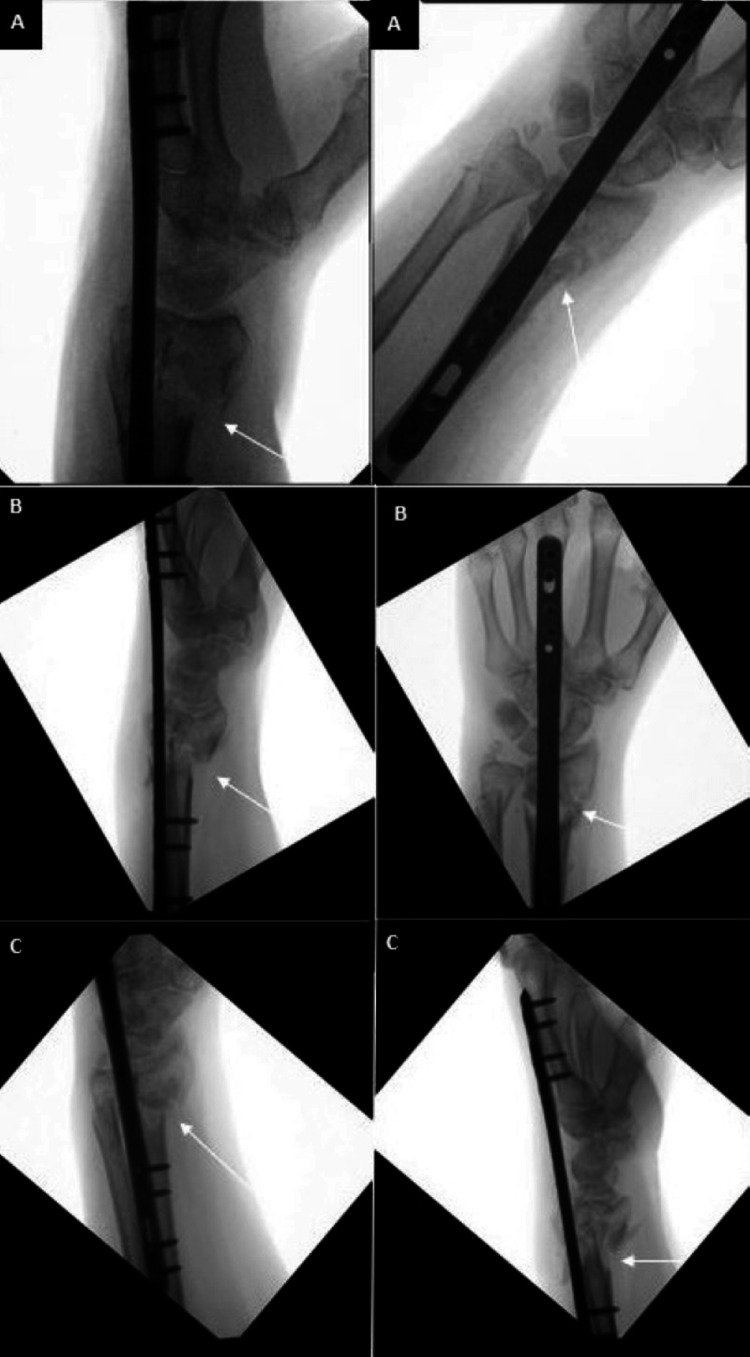
Initial fixation postoperative interval films A: one month; B: two months; C: three months

**Figure 4:**
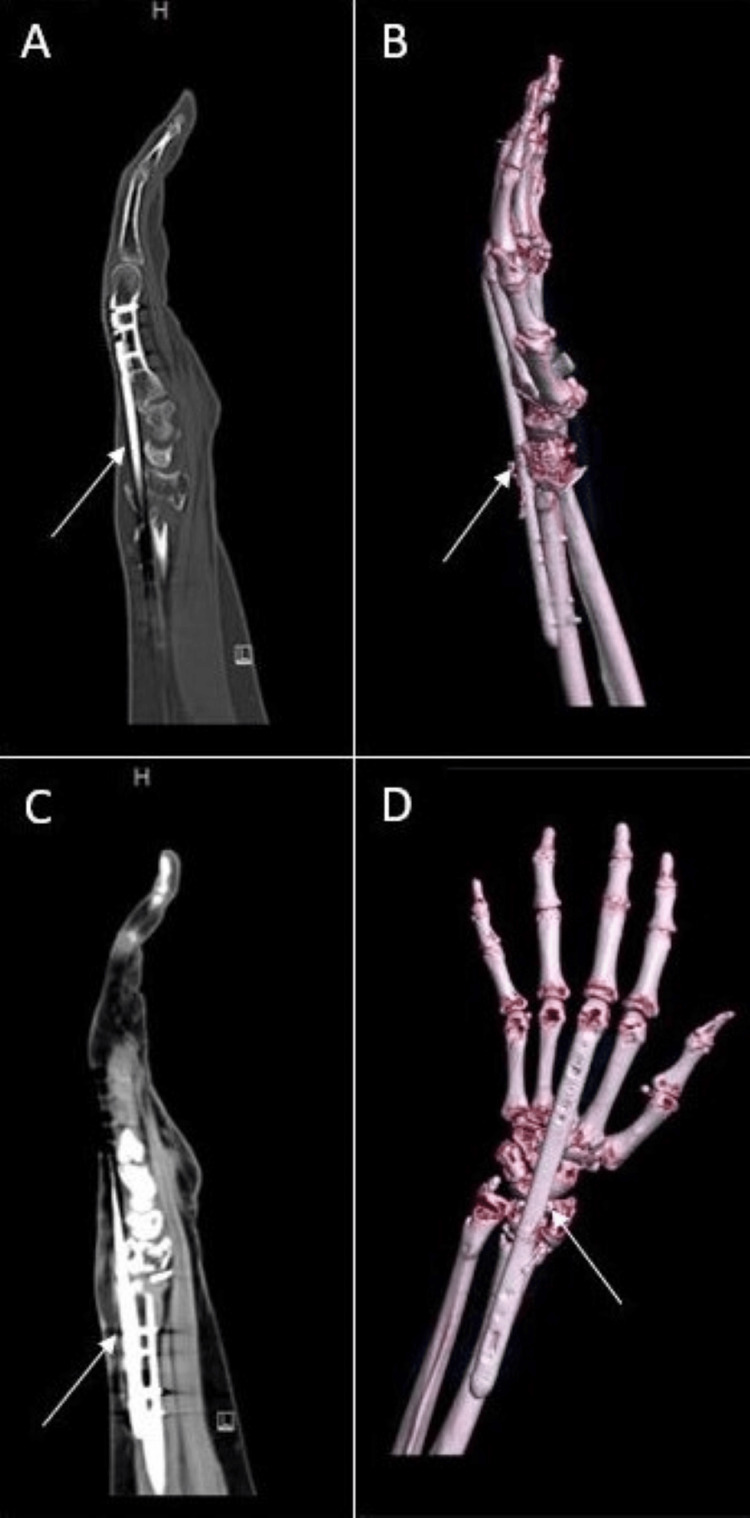
Initial fixation: three-month postoperative films A/C: CT demonstrating the DSP in satisfactory position with fracture healing, but malalignment overall B/D: 3D CT reconstruction films

A 69-year-old female with no history of smoking, alcohol use, or other medical comorbidities, was initially evaluated at an outside hospital for left wrist pain and deformity secondary to sustaining a ground-level fall after being assaulted. She was transferred to our facility for a higher level of care after wrist imaging demonstrated a highly comminuted left distal radius fracture with intra-articular involvement (Figure 1). The on-call physician stabilized this fracture pattern using a short (16 cm) distal radius spanning plate the following morning (Figure 2). After discharge, she was transferred to the Hand Surgery Service for postoperative management. The patient’s alignment appeared reasonable given how comminuted the fracture pattern was, and we decided to evaluate her functional status and healing potential with serial monthly clinical exams and interval imaging (Figure 3A-3C). At three months, a CT scan showed the DSP in a satisfactory position with evidence of fracture healing but with some malalignment overall (Figure 4).

**Figure 5:**
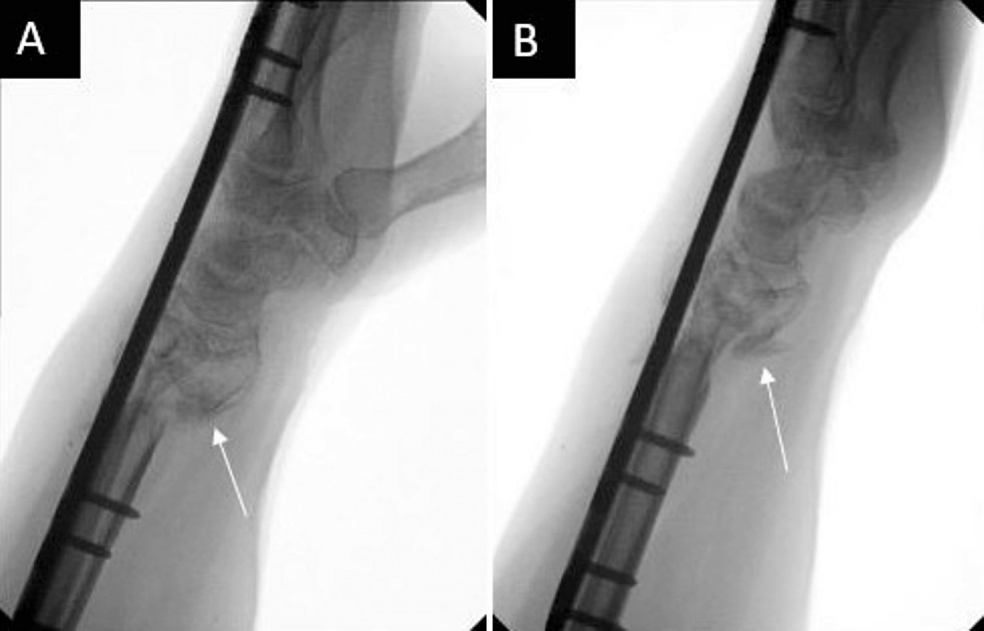
Five-month postoperative films A: Lateral view demonstrating interval callus formation; B: Additional lateral view

**Figure 6:**
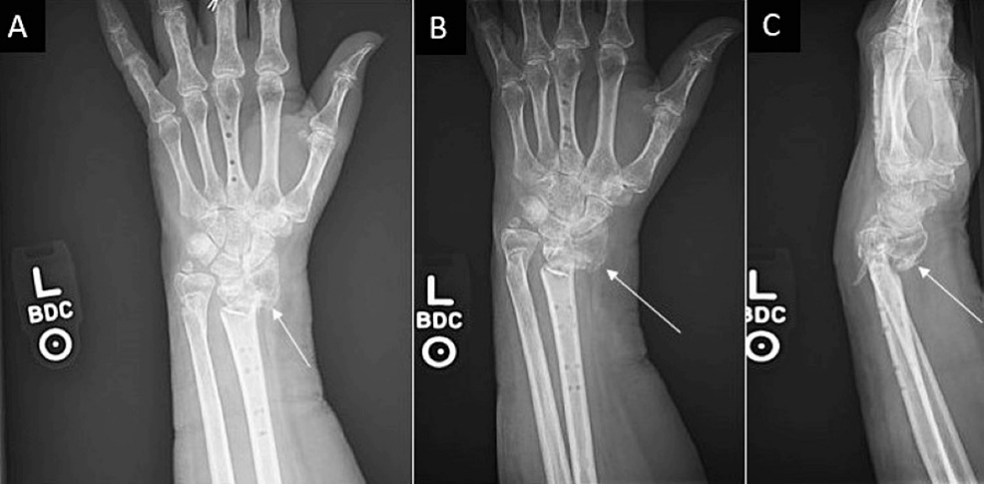
Three-week follow-up imaging after initial spanning plate fixation removal demonstrating fracture site nonunion A/B: AP view; C: Lateral view

**Figure 7:**
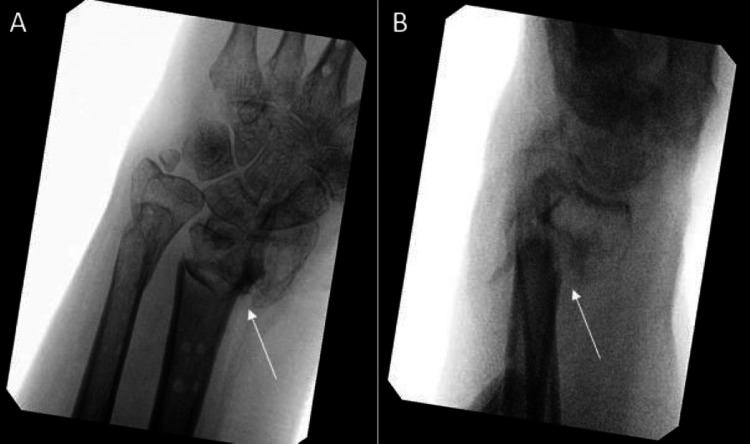
Three-month left wrist films demonstrating nonunion after conservative management A: AP view; B: Lateral view

At five months postoperatively, there appeared to be evidence of bridging callus formation on imaging, and the patient was taken back to the operating room for removal of the spanning plate (Figure 5). Intraoperative fluoroscopy demonstrated distal radius and ulna fractures within tolerance of the patient given her age and history of healing. There was an unchanged alignment of the fracture after removal of the plate, but stress examination under live fluoroscopy demonstrated motion at the fracture site. Given the lack of consent for nonunion treatment, a decision was made to place her into a splint and follow up in the clinic. This was determined after taking into account the patient's age and to allow time for reevaluation of her functional goals. After a discussion of treatment options, the patient wished to continue management with immobilization. Workup of her nonunion, including endocrine, metabolic, and infectious etiologies, found only a significant finding of osteopenia, and the patient was treated with Vitamin D and calcium. Repeat imaging done three weeks later redemonstrated the nonunion and fracture displacement (Figure 6). She was placed into a short arm cast and instructed to use a bone stimulator. After three consecutive monthly follow-ups, interval imaging of the left wrist continued to demonstrate nonunion at the fracture site despite conservative management (Figure 7). The patient endorsed left wrist pain during ROM but denied compressive nerve symptoms. Given these findings, she was scheduled for repeat fixation.

**Figure 8:**
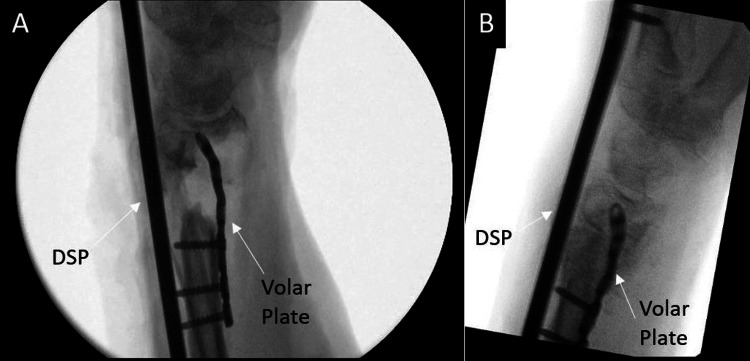
Intraoperative fluoroscopy demonstrating left distal radius fracture fixation using a dorsal spanning plate (DSP), volar intramedullary fragment plate, and viable bone allograft (VBA) in combination with cancellous bone chips A: Lateral view of DSP and volar plate placement, poor subchondral bone stock B: Additional lateral view with good support of the subchondral surface after allograft and bone chips

**Figure 9:**
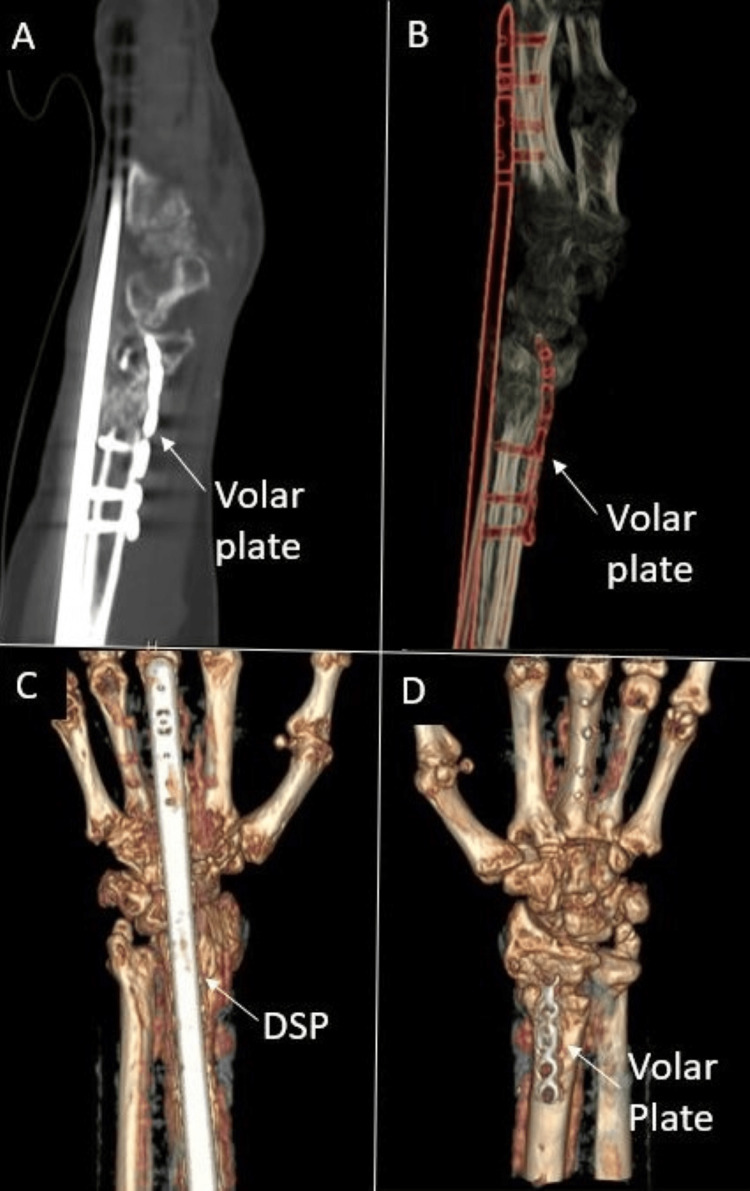
CT left wrist two months status post second fixation A/B: CT films demonstrating dorsal spanning plate (DSP) and volar intramedullary fragment plate placement C/D: 3D CT reconstruction films

Due to notable bone loss at the distal radius fragment, we discussed using an iliac crest bone autograft to support the joint surface. The patient refused this option and nonbiologic allograft alone did not appear feasible given the patient’s poor history of healing. Of the options remaining, the patient agreed to a combination of VBA and cancellous bone chips to support the articular surface. The patient was taken back to the operating room where a long (21 cm) dorsal spanning plate was fixed from the distal radius to the 3rd metacarpal. An intramedullary fragment-specific radial styloid plate was placed over the volar aspect of the distal radius to provide further stability to the subchondral surface. A combination of Cellular Bone Matrix/Allograft and cancellous bone chips mixed in 10 cc of the patient’s blood were used to fill the 2 cm-sized void in the distal radius (Figure 8). A left wrist CT done two months postoperatively confirmed adequate implant fixation and fracture stability (Figure 9).

**Figure 10:**
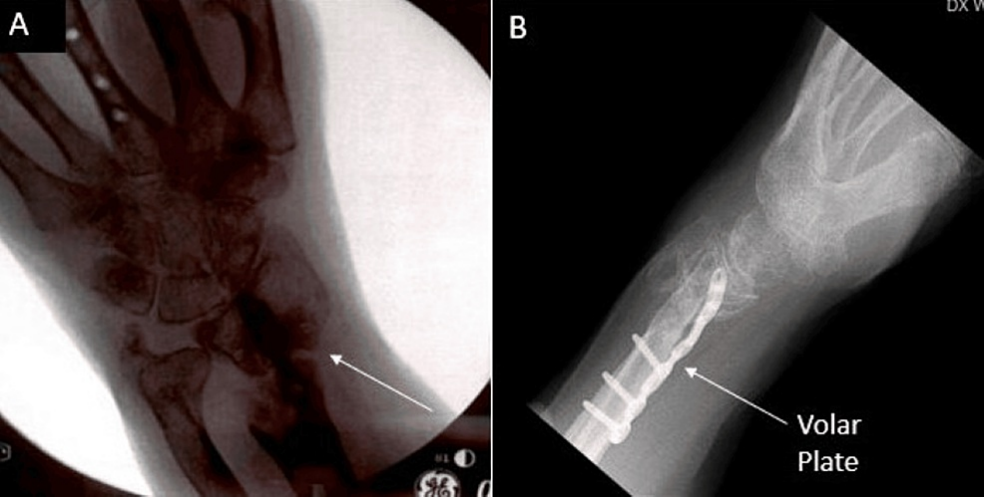
Seven months from the second fixation, the patient’s dorsal spanning plate was removed. The articular surface was well supported after integration using a volar intramedullary plate and a combination of viable bone allograft (VBA) and cancellous bone chips mixed with the patient’s blood A: AP view; B: Lateral view

**Figure 11:**
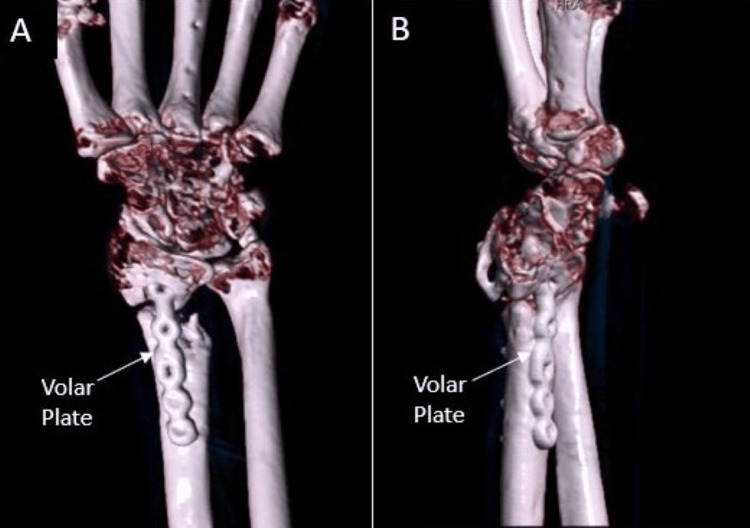
Repeat left wrist CT films done three months after DSP plate removal demonstrated interval callus formation with the hardware positioned appropriately and no violation of the radiocarpal joint A/B: 3D CT reconstructions of left wrist

Seven months from this fixation, the DSP was removed and the intramedullary fragment plate was retained (Figure 10). Intraoperative stress examination under fluoroscopy noted no motion of the fracture site. At clinic follow-up one month later, the patient’s incisions were healing appropriately, and her motor exam was intact. Sensation was intact to light touch at the median, ulnar, and radial distributions of the hand. The following month, the patient advanced to forming a loose fist, but retained stiffness about the 3rd MCP and PIP. There was minimal active wrist flexion and extension, but active supination and pronation were to 60˚ and 80˚, respectively. A repeat left wrist CT scan done three months after DSP plate removal demonstrated good positioning of the intramedullary plate with interval callus formation around the fracture site (Figure 11). The patient was recommended to continue working on ROM exercises at this time. During the latest follow-up done at the one-year mark, the patient denied left wrist pain. Physical exam was significant for continued stiffness with wrist flexion and extension, however active supination and pronation were to 60˚ and 70˚, respectively.

## Discussion

Bridge plating using a DSP has been favorably described for maintaining radial length, distracting the joint surface, and providing rigid stabilization to prevent articular surface loading and subsidence [6,7]. In our patient, alignment after initial stabilization appeared reasonable given extensive comminution, however progression to nonunion led us to design a construct utilizing a DSP, intramedullary fragment-specific plate, and viable bone allograft with cancellous bone chips.

Segalman and Clark described using the amount of subchondral bone supporting the articular surface distal to the nonunion for determining the appropriate treatment of nonunited distal radius fractures [16]. For nonunions with less than 5 mm of subchondral bone of support, they recommended wrist arthrodesis [16]. In their report, they successfully fused six out of nine wrists following this criteria, with four healing after one surgery, one requiring multiple surgeries, and one achieving radiocarpal fusion [16]. In their series, Prommersberger and Fernandez found a high number of postoperative complications in wrist arthrodesis of distal radius nonunions depending on the size of the distal fragment [11]. Given our patient’s poor subchondral bone stock that would not support repair using a volar plate, wrist arthrodesis may have been warranted as a final resort, however, we wished to offer a chance for functional mobility of the wrist while retaining the acceptable alignment seen in our first DSP fixation.

Existing techniques for providing subchondral support in distal radius fractures include auto- or allografting, intramedullary nailing, intramedullary cages, double-tiered subchondral support procedures (DSS) using a variable-angle volar locking plate, or volar peg plating [17-20]. By inserting two screws and two pegs under the subchondral bone of the distal radius, Hardikar et al. demonstrated excellent to good functional results in 85% of patients and a total of 10 mm of subchondral support [17]. For osteoporotic distal radius fractures in elderly patients, Lee et al. found that a DSS procedure and reduction of the distal dorsal cortical distance to 4.6 mm or less adequately maintained length and support [18]. Given our patient’s poor subchondral bone stock that would not support repair using a volar plate and refusal of autograft harvest, we needed a technique that would support the articular surface while satisfactorily providing structural support and stability that would encourage healing. The construct described in this report provided these elements through an intramedullary fragment-specific plate and grafting.

While we acknowledge that this is not an ideal construct for the average patient, this case and patient preferences dictated an unorthodox solution. This technique offered us the opportunity to honor the patient’s autonomy while still providing a stable construct with preserved alignment. The drawbacks of this procedure are that it would complicate future surgical intervention, if needed. Furthermore, additional data is needed to assess whether there are long-term failures associated with our proposed construct.

## Conclusions

In this report, a comminuted intra-articular left distal radius fracture failed to heal with bridge plate fixation. Given the patient’s poor subchondral bone stock and refusal of bone autograft, we designed a construct using a dorsal spanning plate and an intramedullary fragment-specific plate as a volarly placed strut in combination with viable bone allograft and cancellous bone chips to treat this nonunion. This case highlights the importance of ingenuity that orthopedic surgeons should demonstrate when trying to accommodate patients’ wishes as well as creativity that can be portrayed in using implants in a manner unique from their manufactured purpose. Although our patient experienced some postoperative stiffness, she was able to avoid wrist arthrodesis with an acceptable functional outcome at follow-up visits.
